# Standard immunosuppressive treatment reduces regulatory B cells in children with autoimmune liver disease

**DOI:** 10.3389/fimmu.2022.1053216

**Published:** 2023-01-05

**Authors:** Muhammed Yuksel, Farinaz Nazmi, Dima Wardat, Sebahat Akgül, Esra Polat, Murat Akyildiz, Çigdem Arikan

**Affiliations:** ^1^ Paediatric Gastroenterology-Hepatology, Koç University Hospital, Istanbul, Türkiye; ^2^ Liver Immunology Lab, Koç University Research Centre for Translational Medicine (KUTTAM), Istanbul, Türkiye; ^3^ Transplant Immunology Research Centre of Excellence (TIREX) Tissue Typing Lab, Koç University Hospital, Istanbul, Türkiye; ^4^ Paediatric Gastroenterology and Hepatology, Sancaktepe Education and Research Hospital, Istanbul, Türkiye; ^5^ Adult Gastroenterology-Hepatology, Koç University Hospital, Istanbul, Türkiye

**Keywords:** bregs, autoimmunity, liver, children, HLA

## Abstract

**Introduction:**

Autoimmune hepatitis (AIH) is a chronic liver disease caused by a perturbed immune system. The scarcity of short- and long-term immune monitoring of AIH hampered us to comprehend the interaction between immunosuppressive medication and immune homeostasis.

**Methods and patients:**

We recruited children with AIH at the time of diagnosis and at the 1st, 3rd, 6th, 12th, 18th, and 24th months of immunosuppression (IS). We also enrolled children with AIH being on IS for >2 years. Children with drug-induced liver injury (DILI), and those receiving tacrolimus after liver transplantation (LT), were enrolled as disease/IS control subjects. Healthy children (HC) were also recruited. Peripheral blood mononuclear cells (PBMCs) were isolated from all participants. Healthy liver tissue from adult donors and from livers without inflammation were obtained from children with hepatoblastoma. By using flow cytometry, we performed multi-parametric immune profiling of PBMCs and intrahepatic lymphocytes. Additionally, after IS with prednisolone, tacrolimus, rapamycin, or 6-mercaptopurine, we carried out an *in vitro* cytokine stimulation assay. Finally, a Lifecodes SSO typing kit was used to type HLA-DRB1 and Luminex was used to analyze the results.

**Results:**

Untreated AIH patients had lower total CD8 T-cell frequencies than HC, but these cells were more naïve. While the percentage of naïve regulatory T cells (Tregs) (CD4^+^FOXP3^low^CD45RA^+^) and regulatory B cells (Bregs, CD20^+^CD24^+^CD38^+^) was similar, AIH patients had fewer activated Tregs (CD4^+^FOXP3^high^CD45RA**^-^**) compared to HC. Mucosal-associated-invariant-T-cells (MAIT) were also lower in these patients. Following the initiation of IS, the immune profiles demonstrated fluctuations. Bregs frequency decreased substantially at 1 month and did not recover anymore. Additionally, the frequency of intrahepatic Bregs in treated AIH patients was lower, compared to control livers, DILI, and LT patients. Following *in vitro* IS drugs incubation, only the frequency of IL-10-producing total B-cells increased with tacrolimus and 6MP. Lastly, 70% of AIH patients possessed HLA-DR11, whereas HLA-DR03/DR07/DR13 was present in only some patients.

**Conclusion:**

HLA-DR11 was prominent in our AIH cohort. Activated Tregs and MAIT cell frequencies were lower before IS. Importantly, we discovered a previously unrecognized and long-lasting Bregs scarcity in AIH patients after IS. Tacrolimus and 6MP increased IL-10+ B-cells *in vitro*.

## Introduction

Autoimmune hepatitis (AIH) is a fatal immune-mediated liver disorder if left untreated (1). It is characterized by the presence of hypergammaglobulinemia, periportal inflammation with interface hepatitis, and organ (non-)specific autoantibodies. AIH type-1 is defined by the presence of anti-smooth muscle (SMA) and/or anti-nuclear antibodies (ANA), whereas type-2 AIH is diagnosed when liver-specific autoantibodies against Cytochrome P450 enzyme CYP2D6 (anti-LKM1) and/or formimidoyltransferase cyclodeaminase (anti-LC1), are detected (2). Autoantibodies against soluble liver antigens (SLA) can be seen in both types of AIH (3). AIH patients with evidence of cholangitis on histology are diagnosed with autoimmune sclerosing cholangitis (AISC). For more than a half-century, non-selective immunosuppressive drugs (IS) have been used to treat AIH (4). Standard IS in the induction phase exists out of steroids for the first two weeks, and then stacked with azathioprine (AZA) after the second week. Maintenance therapy, depending on patients’ response, side effects, or intolerance, is done with AZA ± steroids, with additional ursodeoxycholic acid in patients with AISC (4). Treatment success is not 100% and is affected by non-adherence, particularly in adolescents, but the type of HLA allele present is also important, as no biochemical remission or frequent relapse (5) was linked with the presence of HLA-DR7 or HLA-DR3 alleles. Furthermore, paediatric AIH is more severe and less controllable compared to adult AIH (6). Hitherto, a detailed systemic and hepatic profile of the activity of innate and adaptive (regulatory) immunity, prior to the start of any IS, has been elusive. The general concept of the AIH immunopathophysiology is that loss of self-tolerance of autoreactive T cells results in T helper (TH) 1, TH2, and TH17 cells, accompanied by regulatory T cell aberrations (2, 7, 8), stimulating CD8 T cells, B cells and NK cells to ultimately damaging hepatocytes *via* cellular, humoral, and granzyme mediated auto-aggression, respectively (9). By virtue, the use of IS is the mainstay of AIH treatment. Although IS improves hepatitis in the majority of patients, it also suppresses Tregs, the very cells that constitute immune tolerance. More importantly, Diestelhorst et al. clearly demonstrated that standard IS treatment reduced inflammation (CD4/CD8 cell infiltration) by 39% (10). Yet, the proportion of Tregs (CD4^+^FOXP3^+^) was disproportionally diminished by more than 50%, which may explain why weaning off IS is not justified given our current and partial comprehension of AIH pathophysiology at baseline and after IS. T cell biology has received a lot of attention, but B cells are also very important because bidirectional B cell-T cell communication is crucial in both homeostasis and disease. For example, regulatory B cells (Bregs) interact with Tregs, causing an increase in their numbers (11). However, little is known about Breg homeostasis in AIH. To that end, our primary aim was to investigate the immunological properties of naïve (untreated) and treated AIH patients using deep immune phenotyping of PBMCs and intrahepatic immune cells. The secondary aim was to understand the B cell dynamics and effects of IS drugs on B cells and Bregs, as well as whether there is a difference between type-1 and type-2 AIH. Lastly, we also investigated the HLA-DRB1 allele frequencies in these patients in order to better understand the pattern of HLA alleles associated with AIH patients.

## Patients and methods

### Study population

The study protocol was approved by Koç University (IRB: 2019.255.IRB2.077 and 2019.262.IRB2.084) and complied with the Declaration of Helsinki’s ethical principles from 1975. Written informed consent was obtained from all parents and/or patients. Between 2019 and 2022, we recruited children for the different study groups. Group-1 consisted of naïve AIH patients with sequential follow-up samples after the standard IS regimen, consisting of Pred and Aza as per the guidelines (4, 12); Group-2 AIH patients are cross-sectional ones under treatment, and Group-3 AISC patients are also cross-sectional ones being treated. Group-4 children are recruited as age-matched healthy controls (with only functional constipation), Group-5 are patients with drug-induced liver injury (DILI), and Group-6 have had a liver transplant >6 months ago, have a protocol liver biopsy, and are treated with tacrolimus. Group-7 includes patients who underwent a partial hepatectomy due to hepatoblastoma, and Group-8 includes donors that had undergone surgery as living-related liver donors for their relatives/next of kin (2019.020.IRB2.019). **Table 1** lists the demographics for each group, and **Supplementary Figure 1** shows a schematic overview of each group. We collected the following data: gender, age, autoantibodies anti-nuclear antibody (ANA), anti-smooth muscle antibody (ASMA), anti-soluble liver antibody (SLA), anti-mitochondrial antibody (AMA), liver kidney microsomal type-1 antibody (anti-LKM1), and liver cytosol antibody type 1 antibody (anti-LC1), perinuclear-anti neutrophil cytoplasmic antibodies (pANCA), alanine aminotransferase (ALT), aspartate aminotransferase (AST), gamma-glutamyl transferase (GGT), immunoglobulin G (IgG) (**Table 1**) from patient charts and electronic patient records. The diagnosis of AIH was made according to EASL AIH guidelines (12).

**Table 1:** Demographics and clinical parameters.

GROUPS Number (n)	AGE Months (IQR)	GENDER F/M	ALT<41IU(IQR)	AST<52IU(IQR)	GGT<18IU(IQR)	IgG5-15 g/L (IQR)	Presence of Autoantibodies
**Group-1:** **AIH** **NAIVE PATIENTS** **(7)**	103 (50-221)	4/3	180 (61-1119)	64 (26-697)	49 (25-116)	15.8 (10-17)	Anti-ANA/SMA: 28.5% (2) Anti-LKM1/LC1: 57% (4) Seronegative: 14.3% (1)
**Group-2: AIH LONGTERM FOLLOW-UP** **(9)**	192 (142.5-212)	5/4	34 (32-50)	38 (34-51)	22 (13-29)	13.7 (10.8-14.9)	Anti-ANA/SMA: 89.9% (8) Anti-LKM1/LC1: 0% (0) Seronegative: 11.1% (1)
**Group-3: AISC** **overlap** **(3)**	204 (177-213)	1/2	34 (15-63)	34.5 (18-51)	51 (28-88)	10.8 (7.7-14)	Anti-ANA/SMA: 66% (2) pANCA: 100% (3)
**Group-4: HC** **PBMC** **(7)**	91 (57-108)	2/5	14 (13-15)	25 (na)	15 (na)	9.56 (7.8-10.6)	na
**Group-5: DILI** **(3)**	144 (52-204)	1/2	386 (15-1457)	97 (15-122)	179 (14-413)	8.8 (7.6-9.6)	0%
**Group-6: LT** **(9)**	72 (24-102)	5/4	41 (25-77)	46 (37-67)	25 (13-68)	12.9 (8.1-15.4)	na
**Group-7: HBL** **(3)**	35 (26-174)	2/1	32 (27-409	50 (50-290)	24 (20-62)	7.3 (4.9-13.9)	na
**Group-8: HC DONOR** **LIVER** **(8)**	412 (262-444)	5/3	13 (11-23)	17 (14-22)	16 (13-24)	na	na

DILI, drug-induced liver injury; HBL, hepatoblastoma; PBMC, peripheral blood mononuclear cells; LT, liver transplanted patients; ALT, alanine aminotransferase; AST, aspartate aminotransferase; GGT, gamma glutamyl transpepdidase; IQR, interquartile range; IgG, immunoglobulin G; anti-ANA, anti-nuclear antibodies; anti-SMA, anti-smooth muscle antibody; anti-LKM1, anti liver kidney microsomal antibodies; anti-LC1, anti liver cytosol antibody; pANCA, perinuclear antineutrophil cytoplasmic antibodies; AIH, autoimmune hepatitis; AISC, autoimmune sclerosing cholangitis; na, not applicable.

### Liver biopsy and fine needle aspiration

Children that underwent a liver biopsy, either for diagnostic or routine histological assessment, conform to the guidelines (13). The biopsy and fine needle aspiration (FNA) procedures were both ultrasound guided under general and local anesthesia. As we recently published, FNA was used to obtain intrahepatic lymphocytes (IHL) (14). The aspirate was then filtered through a 40μm filter and collected in cold phosphate-buffered saline (PBS) containing 2% FBS (Biowest) (14).

### Peripheral blood mononuclear cells isolation, cell staining, and gating strategy

**Figure 1:**
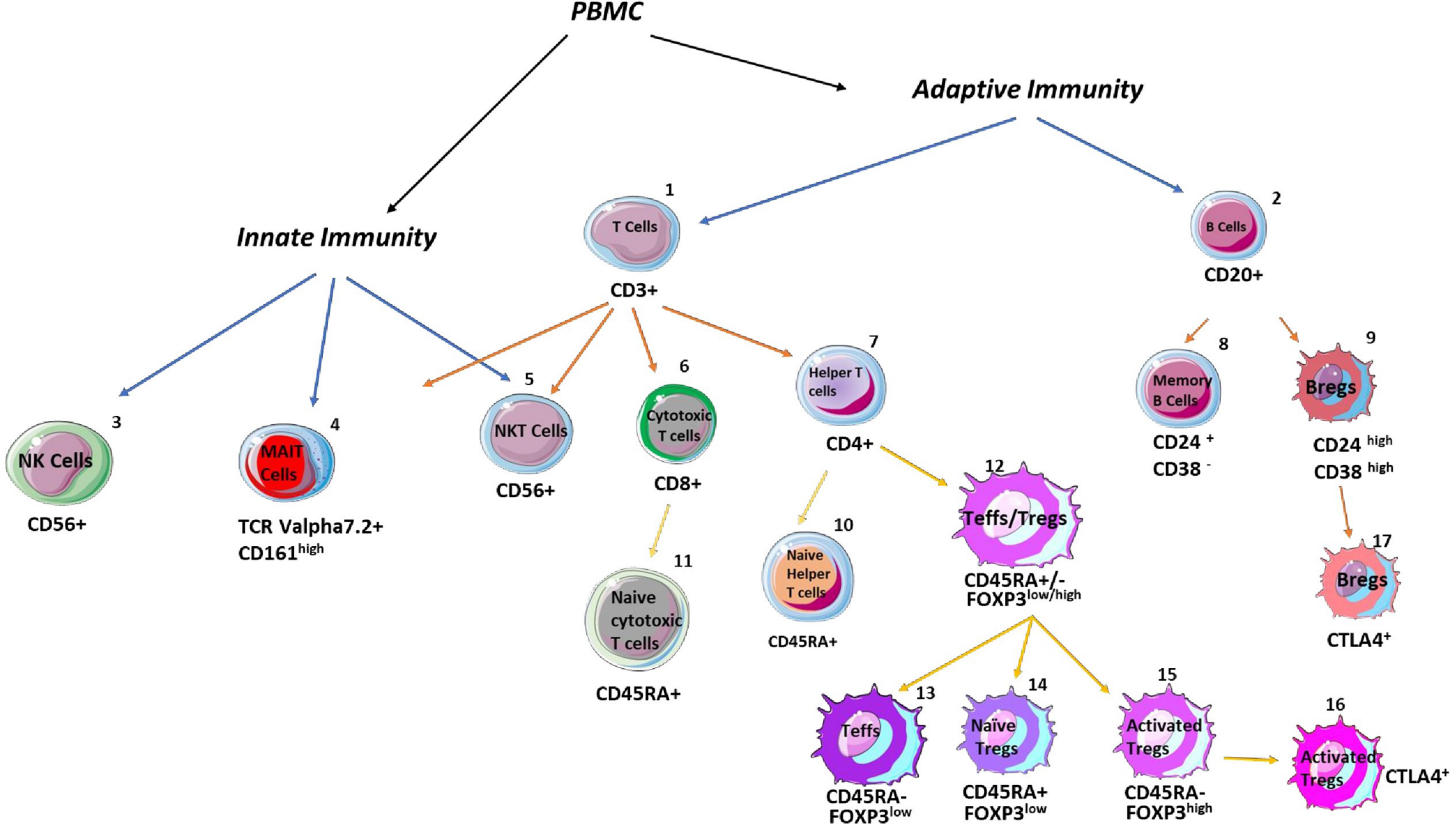
Schematic overview of analysed immune cells with their specific markers. The percentages of adaptive and innate immune cells were assessed in the blood and livers. The following CD markers were applied to define total T cells: CD3+CD20-, T helper cells: CD4+CD8-, cytotoxic T cells: CD8+CD4-, naïve T helper cells: CD4+CD45RA+, naïve cytotoxic T cells: CD8+CD45RA+, naïve regulatory T (Tregs) cells: CD4+CD45RA+FOXP3low, activated Tregs: CD4+CD45RA-FOXP3high, CTLA-4 positive Tregs: CD4+CD45RA-FOXP3highCTLA-4+, total B cells: CD3-CD20+, memory B cells: CD20 +CD24+CD38-, regulatory B (Bregs) cells: CD20+CD24highCD38high, CTLA-4 positive Bregs: CD20+CD24highCD38high, NK cells: CD3-CD56+, NKT cells: CD3+CD56+, and mucosal-associated invariant T cells: CD3+CD161+TCRVα7.2+.

To isolate peripheral blood mononuclear cells (PBMC), whole blood was diluted with PBS and layered onto Lymphoprep (GE), followed by density gradient centrifugation (15). Following a PBS wash, cells were resuspended in PBS, stained with viability dye EF780 (BD-eBioscience, ThermoFisher) before being incubated with cell surface antibodies specific for (regulatory) T-cells, mucosal-associated-invariant-T-cells (MAIT), (regulatory) B cells, and NK/NKT cells as previously described (7, 14–16). These antibodies are; CD3 (clone HIT3a), CD161 (clone HP-3G10), CD20 (clone 2H7), CD24 (clone ML5), CD25 (clone M-A251), CD38 (clone HIT2), CD4 (clone A161A1), CD45RA (clone HI100), CD56 (clone 5.1H11), CD8 (clone HIT8a), HLA-DR (clone L243), and TCR Valpha7.2 (clone 3C10) (**Figure 1**), and gating strategy is shown in **Supplementary Figure 2**. FOXP3 was determined by intranuclear staining with anti-FOXP3 (clone PCH101, eBioscience) and intracellular CTLA-4 was detected with anti-CTLA-4 (clone BNI3, Biolegend) after FOXP3 cellular fixation/permeabilization buffer (eBioscience) was used, followed by addition of intracellular permeabilization buffer (eBioscience). After washing with PBS, the cell pellet was resuspended in 300μL of PBS and passed through a multicolor flow cytometer (Attune-NxT, ThermoFisher). Analysis was performed using Flowjo (Treestar Inc, USA).

### Cell stimulation and *in vitro* immunosuppression with drugs

The PBMCs were first resuspended in complete cell medium (RPMI-1640 with L-glutamine and 0.5% penicillin/streptomycin (GIBCO), 0.5% amphotericin (Sigma-Aldrich), and 10% heat-inactivated fetal bovine serum) (GIBCO). These cells were then seeded onto a 96-well round-bottom plate at a density of 200,000 cells/200μL/well. The *in vitro* effects of IS drugs were explored by adding prednisolone, the active metabolite of azathioprine (6-MP), rapamycin, or tacrolimus (Sigma-Aldrich), at a final concentration of 10 nM, according to the published protocol (17). After 2 hours of incubation with IS drugs (18), cells with and without IS were stimulated for 6 hours at 37°C in the incubator with a cell activation cocktail (Biolegend) and recombinant human IL-2 (Biolegend) (100U/ml) (2). For intracellular cytokine staining, PBMCs were stained with cell surface antibodies staining B cells (CD20) and Bregs (CD20CD24CD38). Cells were fixated for 20 mins, washed with FBS, and the cell pellet was resuspended with intracellular permeabilization buffer (eBioscience), followed by anti-IL-10 (clone JES3-19F1) antibody incubation for 30 mins at 4°C. Thereafter, cells were washed with PBS and resuspended in 300μL of PBS, prior to flow cytometry.

### HLA determination

The routine tissue typing lab at Koç University Hospital used the Lifecodes HLA-DR eRES SSO typing kit (ref:628925) to genotype HLA DRB antigens. Data were collected on a Luminex platform, and allele allocation was performed by a trained lab technician using MATCH IT! DNA software.

### Statistics

Statistical analysis was performed using GraphPad 5. Normality was assessed with Kolmogorov-Smirnov Test. Comparisons between two groups were performed by student’s t-test or Mann-Whitney U test. Multiple comparisons were analyzed by one-way analysis of variance or by Kruskal-Wallis, depending on the normality. For parametric variables, the standard error of the mean ( ± SEM) was provided, whereas, for non-parametric variables, we used the interquartile range (IQR). A p-value of <0.05 was considered significant.

## Results

### Peripheral blood CD8 T cells, activated Tregs, and MAIT cells were lower in treatment-naïve autoimmune hepatitis patients, compared to healthy controls

**Figure 2:**
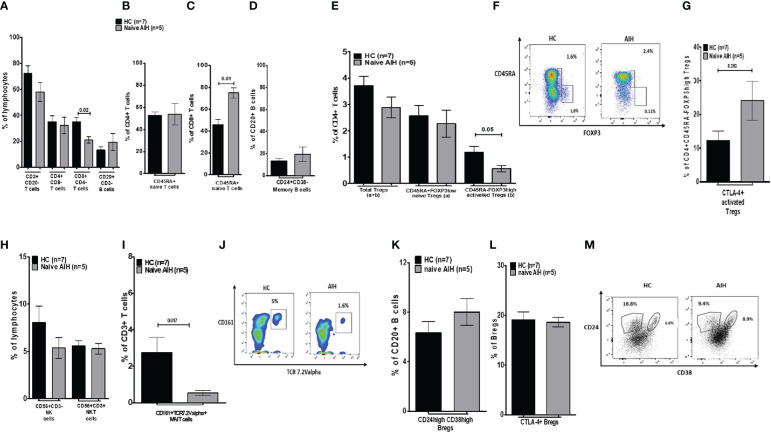
Analysis of the distribution of lymphocytes and their subsets in blood, in healthy controls and naïve autoimmune hepatitis patients. **(A)** Is a bar chart of the frequency of peripheral blood immune cells such as CD3, CD4, and CD8 T cells, and total CD20 B cells. **(B, C)** Show naïve CD4 and CD8 T cells. **(D)** Shows memory B cells. **(E)** Demonstrates the proportion of total, naïve, and activated Tregs. **(F)** Is a depiction of gating for Tregs. **(G)** Provides frequencies for CTLA-4+ Tregs. **(H)** Is a depiction of NK/NKT cell proportion. **(I)** Shows MAIT cells and **(J)** is showing the gating for MAIT cells. **(K)** Is a bar chart of Bregs and **(L)** shows CTLA-4 positive sub-group. **(M)** Depicts gating for memory B cells and Bregs. Five naïve AIH patients and seven healthy controls were included for this part of the study.

As depicted in **Figure 1** and **Supplementary Figure 2**, we have investigated 17 cells (and subsets) in the peripheral blood of AIH patients. The frequency of CD3+ T cells, CD20+ B cells, CD4+ T cells, and naïve CD4 T cells was unaltered compared to PBMCs of HCs (**Figures 2A, B**). Total CD8+ T cells (21.1%) were significantly lower compared to HCs (35%), whereas these cells were more naïve compared to their counterparts (74.9% versus 46%) (**Figures 2A, C**). CD24+CD38- memory B cells were unaltered (**Figure 2D**). In terms of Treg homeostasis in peripheral blood, we found fewer activated Tregs (CD4^+^FOXP3^high^CD45RA**^-^**) in treatment-naïve AIH patients than in HCs (0.56% versus 1.18%) (**Figures 2E, F**). Albeit, the proportions of naïve (CD4^+^FOXP3^low^CD45RA**^+^**) and total (activated + naïve) Tregs were comparable between AIH and HCs. Furthermore, the ratio of total Tregs to effector T cells (Teffs: CD4^+^FOXP3^low^CD45RA**^-^**) were similar between AIH patients (**Supplementary Figure 3A**) and HCs. Additionally, activated Tregs from treatment-naïve AIH patients expressing CTLA-4 were similar compared to HCs (24.1% versus 12.5%) (**Figure 2G**).

We also explored the proportions of innate immune cells such as MAITs (CD3+CD161+Vα7.2+), NK (CD3-CD56+), and NKT (CD3+CD56+) cells. Even though NK/NKT cell frequencies were indifferent between treatment-naïve AIH and HCs, MAIT cell frequencies in treatment-naïve AIH were much lower (0.54%) compared to HCs (2.7%) (**Figures 2H–J**).

Furthermore, treatment-naïve AIH patients had similar proportions of Bregs (CD20^+^CD24^high^CD38^high^) in comparison to HCs (8% versus 6.3%) (**Figures 2K–M**). A significant proportion of Bregs expresses CTLA-4 (19). CTLA-4+ Bregs from treatment-naïve AIH (18.6%) were equally abundant as HC Bregs expressing CTLA-4 (19.1%) (**Figures 2L, M**).

### Immunophenotypic monitoring following IS treatments in the prospective group

**Figure 3:**
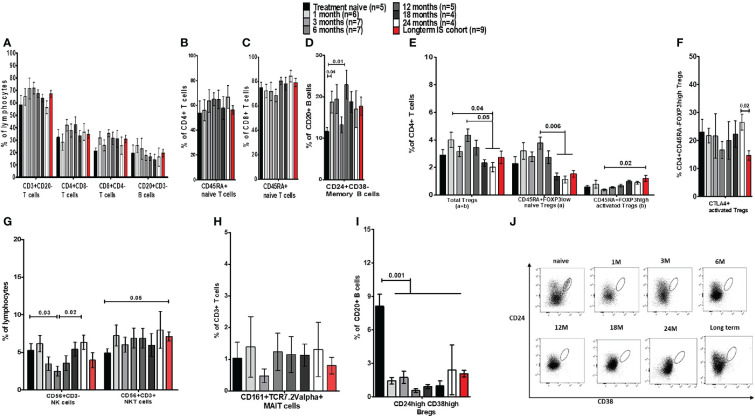
Frequency analysis of lymphocytes and their subsets in blood before and during immunosuppressive treatment of autoimmune hepatitis. **(A)** Is a bar chart of the frequency of peripheral blood immune cells such as CD3, CD4, and CD8 T cells, and total CD20 B cells from AIH patients who were either prospectively followed up from prior to IS until 24 months during treatment (prospective cohort) or assessed cross-sectionally (long-term cohort). **(B-D)** Show naïve CD4 and CD8 T cells, and memory B cells in these patients. **(E)** Demonstrates the proportion of total, naïve, and activated Tregs. **(F)** Provides frequencies for CTLA-4+ Tregs. **(G)** Provides frequencies of NK/NKT cells and **(H)** shows MAIT cells. **(I)** Is a bar chart of Bregs in these AIH patients. **(J)** Depicts gating for Bregs analysed at different time points.

The peripheral immune composition in the prospective treatment group was assessed after 1, 3, 6, 12, 18, and 24 months of standard IS therapy. We observed that the total T cells (CD3+), total B cells (CD20+) as well as CD4 T – and CD8 T cell frequencies, remained stable over the course of 24 months (**Figure 3A**). Regarding the activation status (CD45RA expression), the proportion of naïve CD4 T and CD8 T cells remained stable as well (**Figures 3B, C**). Nonetheless, memory B cells (CD20^+^CD24^+^CD38**^-^**) significantly increased from 11.6% at diagnosis to 18.7% at 1 month and 22.9% at 12 months, remaining somewhat high thereafter (**Figure 3D**).

### Activated Tregs increased in long-term follow-up patients whereas Bregs remained low

The cross-sectional cohort who were >2 years on IS broadly followed the immune phenotypical trends observed in the prospective cohort (**Figures 3A–D**).

Next, we also investigated whether short/long-term IS affected Treg homeostasis. We demonstrated that the proportion of total Tregs fluctuated non-significantly for several months after IS. Nonetheless, the percentage of total Tregs fell at months 18, 24, and thereafter when compared to earlier time points. This was also observed in the naïve Tregs population, which was significantly less than 1.5% in the last three time points, compared to the first cohort’s month 6 (3.7%) of IS treatment (**Figure 3E**). On the contrary, activated Tregs were significantly higher (1.17%) in the long-term IS group compared to month 3 of IS (**Figure 3E**). However, the proportion of activated Tregs that expresses CTLA-4 showed a significant reduction in the long-term group (**Figure 3F**). Furthermore, the ratio of Teffs to total Tregs in AIH patients was significantly higher than in HCs (**Supplementary Figure 3A**), indicating an unresolved immune dysregulation.

We also investigated the frequencies of NK/NKT cells and MAIT cells (**Figures 3G, H**). NK cells exhibited significant fluctuations during the different time points. NKT cell proportion was higher in the long-term cohort compared to baseline levels, and MAIT cell frequencies remained unaltered. Although the frequency of Bregs was 8.1% prior to treatment, following IS therapy, we observed a substantial reduction in Bregs to 0.75% at 6 months (**Figures 3I, J**). In the long-term cohort, Bregs remained significantly low (<2.5%) compared to treatment-naïve patients (**Figures 3I, J**). The expression of CTLA-4 on the few remaining Bregs could not be assessed due to the low number of events (data not shown).

In terms of comparisons between AIH-1 and AIH-2, we only found that at 3 months of IS, the frequency of NK cells was higher in AIH-2 (2.9%) than in AIH-1 (1.7, p=0.05). All other cell populations were similar (data not shown). The difference was unrelated to IS because all AIH-1 and AIH-2 patients received Aza+Pred at that time. Furthermore, AIH patients on long-term IS were compared to AISC patients and there were no differences observed between the two groups (**Supplementary Figures 4A–I**).

### More B cells, and fewer Tregs and Bregs in autoimmune hepatitis patients’ liver

**Figure 4:**
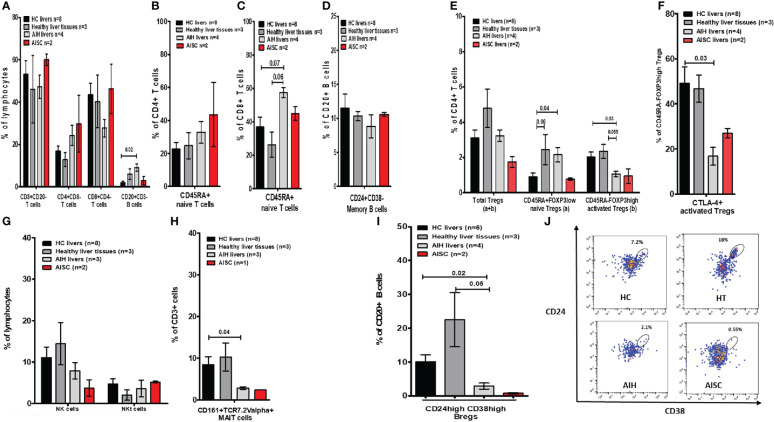
Frequency analysis of intrahepatic lymphocytes in autoimmune hepatitis, autoimmune sclerosing cholangitis, healthy donor livers, and age-matched healthy liver tissue. **(A)** Is a bar chart of the frequencies of intrahepatic immune cells such as CD3, CD4, and CD8 T cells, and total CD20 B cells. **(B, C)** Show naïve CD4 and CD8 T cells. **(D)** Shows memory B cells. **(E)** Demonstrates the proportion of total, naïve, and activated Tregs. **(F)** Provides frequencies for CTLA-4+ Tregs. **(G)** Is a depiction of NK/NKT cell proportions. **(H)** Shows MAIT cells. **(I)** Is a bar chart of Bregs and **(J)** shows gating for Bregs.

Following the immune profiling of PBMCs, we also aimed at elucidating the intrahepatic immune microenvironment. Intrahepatic lymphocytes were isolated from FNAs, during liver biopsies for the diagnosis of AIH (n=1) or the follow-up of AIH (n=3) or AISC (n=2). In contrast to CD3 T cells, we found that CD20 B cells were significantly enriched in the livers of AIH patients (9.0%) compared to HCs (2%) (**Figure 4A**). Other cell populations such as CD4 and CD8 T cells, and their CD45RA expressing subsets, as well as memory B cells, were similar (**Figures 4A–D**).

In terms of the intrahepatic Treg (sub)population, we found that naïve Tregs were more prevalent in AIH than HC. When compared to HCs (2%) and HTs (2.3%), the frequency of activated Tregs was significantly lower (1.0%) (**Figure 4E**). Furthermore, the Teffs/Tregs ratio in AIH livers was comparable between the groups (**Supplementary Figure 5A**). Additionally, the proportion of activated Tregs expressing CTLA-4 was significantly lower when compared to HC (**Figure 4F**). Moreover, the proportions of NK and NKT cells were unaffected, whereas the frequency of MAITs cells was significantly lower in AIH livers (2.9%), compared to HCs (8.5%) and HTs (10.3%) (**Figures 4G, H**). Complementary to what we saw in peripheral blood, the proportion of intrahepatic Bregs in AIH livers (2.9%) was significantly lower than in HCs (10%) and HTs (22.3%) (**Figures 4I, J**).

**Figure 5:**
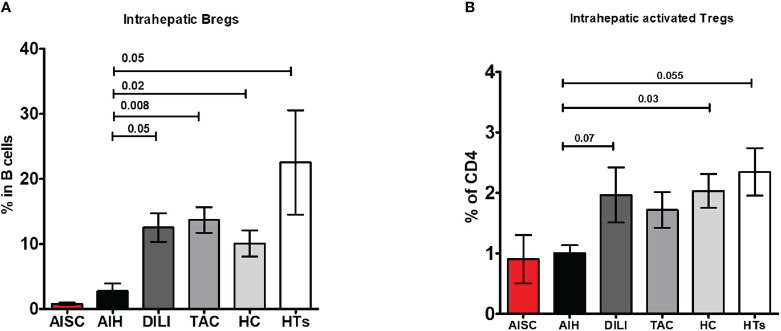
Frequency analysis of intrahepatic regulatory B cells and activated regulatory T cells. **(A, B)** Are bar graphs of intrahepatic Bregs and activated Tregs, respectively, in patients with AISC (n=2), AIH (n=4), drug-induced liver injury (DILI) (n=3), liver transplanted patients on tacrolimus monotherapy (n=9), healthy donor livers (n=6) and healthy tissue from hepatoblastoma patients (n=3).

To determine whether the paucity of liver Bregs is caused by disease or treatment, we compared the intrahepatic proportion of AIH Bregs to the intrahepatic proportion of DILI (disease control) and transplanted children on tacrolimus. We found that both DILI and post-transplant patients on tacrolimus had significantly more intrahepatic Bregs, 12.9% and 13.6% respectively, than AIH patients (**Figure 5A**). Similarly, we wondered if the low proportion of intrahepatic Tregs was unique to AIH, or a result of treatment and on comparison, found out that intrahepatic activated Tregs were in fact similar in AIH and DILI (**Figure 5B**).

### Effect of immunosuppressive drugs on regulatory B cells

**Figure 6:**
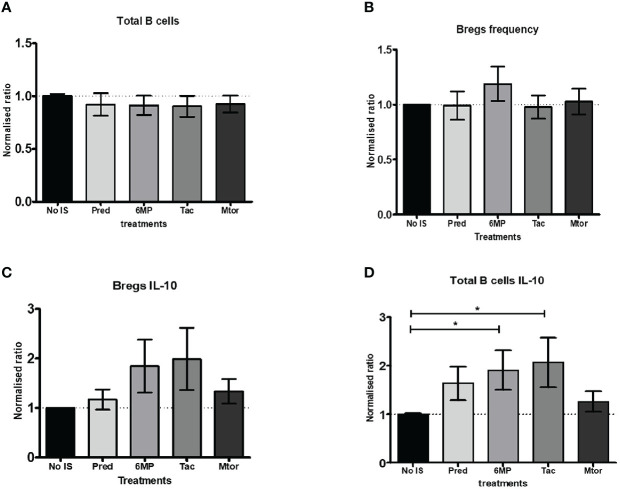
Analysis of *in vitro* effects of immunosuppressive drugs on B cells and Bregs producing IL-10. **(A, B)** Show bar charts of normalised total B cells and Bregs frequencies following incubation with medium only or with additional (10 mM) immunosuppressive drugs such as prednisolone, 6MP (active form of azathioprine), tacrolimus, or rapamycin (Mtor-inhibitor). **(C, D)** are bar charts of normalised total B cells and Bregs frequencies producing IL-10 under the same circumstances as in **(A, B)**. The PBMCs from seven (age-matched) healthy controls were included in this part of the study. *, 0.05.

Finally, we wanted to know which IS drug in particular affected the frequency of Bregs. However, because we were short of PBMCs from naïve AIH patients, we used blood from HCs. We measured the frequency of total B cells, IL-10+ B cells, Bregs, and IL-10+ Bregs in PBMCs after incubating them with various IS drugs followed by a cytokine stimulation cocktail. In these *in vitro* conditions, the normalized ratios of total B cells and Bregs frequencies did not differ from baseline (no IS) levels (**Figures 6A, B**). Furthermore, when 6MP and Tac were used, the normalized ratio of IL-10 producing Bregs tended to be higher than at baseline (**Figure 6C**). Nonetheless, we found that 6MP and Tac significantly increased the normalized ratios of IL-10+ total B cells (**Figure 6D** and **Supplementary Figure 6C**).

### HLA-DR11 allele was prominent in AIH patients

Lastly, we also explored the frequency of HLA-DRB1 alleles in AIH patients. We found that HLA-DR11 was the most frequent allele in AIH-1 (70%) and AIH-2 (66%) patients (**Supplementary Figures 7A, B**). Alleles known to be associated with AIH in Caucasian AIH patients (DR3, DR4, DR7, DR13) had lower frequencies (**Supplementary Figures 7A, B**). We were also able to investigate the presence of the HLA-DRB1 allele in two AISC patients. One was DR3 homozygous, while the other was DR15 homozygous. We also compared ALT, AST, GGT levels, and immune profiles in DR11+/- patients but found no differences (data not shown). Due to the small sample size, it was not possible to assess patients’ responses to IS (early remission, good response, or frequent relapse) according to their HLA-DRB1 allele.

## Discussion

The aetiopathology of AIH is only partially understood. It is characterized primarily by the activation of adaptive immunity and an aberration in immunoregulatory mechanisms. While T-cells play a central role in the pathogenesis by producing pro-inflammatory cytokines coinciding with Tregs alterations, data concerning the role of B-cells is elusive. However, their potential role could be important as B cells also contain a variety of immunoregulatory B cell subtypes such as CD24CD38 double-positive Bregs producing anti-inflammatory cytokines IL-10 and IL-35. Therefore, in this study, we reported on the findings of immune phenotyping of T, NK, NKT, and MAIT cells as well as B cells and their subtypes in treatment naïve, short- or long-term IS drug-treated children with AIH.

In essence, in the peripheral blood of naïve AIH patients, only total CD8 T cells and MAIT cells were significantly lower, whereas naïve CD8 T cells were more abundant compared to HCs. Regarding the immunoregulatory immune cell compartment, total Tregs and naïve Tregs were equally distributed in AIH and HCs. Yet, activated Tregs were lower in AIH compared to HCs. Importantly, the proportion of Bregs and CTLA-4+ Bregs was unaltered. After beginning IS medications, there were fluctuations, but only the frequency of memory B cells changed significantly from the starting point. Following the start of IS, there were marginally higher Treg frequencies (total and naïve), which subsequently started to decline after 18 months of IS in comparison to earlier time points. However, one cell population did not recover from IS treatment; the proportion of Bregs dropped sharply after IS and remained low throughout the entire investigation. Due to non-adherence, Bregs only returned in one patient (6.9%), which accounts for the significant variation at the 24-month mark. Regarding the hepatic immune microenvironment, naïve CD8 T cells and total B cells were higher in AIH. Furthermore, not total nor naïve but activated Tregs in AIH were lower than HCs. In AIH livers, MAIT cells were also less frequent. Yet again, Bregs were lower in AIH livers.

Few studies have been conducted to date, mostly on adults, to better understand how IS medications affect systemic and/or intrahepatic lymphocytes in patients with AIH (3, 10, 20–22). Most notably, by means of immunohistochemistry (IHC), Diestelhorst et al. noted a sharp decline (>50%) in intrahepatic Tregs proportions, following IS in children with AIH. However, it is unclear how this decline will affect long-term treatment outcomes in these children. The same group also investigated hepatic Treg homeostasis in adult AIH (20). There, they found that following IS, hepatic Tregs were reduced, especially in patients with incomplete remission (IR). Importantly, the baseline (treatment naïve) hepatic Treg proportions in these IR patients were not lower compared to patients that would become biochemical responders (BR) after IS. This demonstrates that IR is not necessarily the consequence of any initial Treg numerical impairment, at least when compared to the BR patients. However, as they used IHC, further delineation of Tregs subtypes was not possible. Our flow cytometric analysis of intrahepatic Tregs demonstrated that the frequency of total Tregs was indifferent compared to HCs and age-matched HTs. Contrarily, naïve (Sakaguchi type I Tregs (16)) Tregs were higher in AIH livers only when compared to adult livers. Yet, Sakaguchi type II Tregs (16) (activated Tregs) were lower in AIH compared to both HC/HT livers and expressed less CTLA-4. This supports the notion that a detailed Tregs subgroup analysis and selecting appropriate control groups are incumbent for a valid comparison. Additionally, this is the first report of CTLA-4 expression on intrahepatic Tregs. Nonetheless, one recent paper did report the intrahepatic CTLA-4 expression on total CD4 T and CD8 T cells within the liver. The proportion of CTLA-4 was similar between normal livers and adult AIH livers, whether they were under treatment or not (22).

Multiparametric flow cytometry also permitted us to investigate unconventional T cells, such as MAIT cells. In blood, the proportion of MAIT cells was lower at baseline and did not recover after IS treatment in pediatric AIH. Hepatic MAIT cell frequency was also low compared to HC. Similar results were noted recently, by two unrelated investigations in adult AIH patients (3, 23). Moreover, they found that these MAIT cells expressed similar or higher levels of granzyme-B, but similar proportions of IFN-γ and TNF-α were detected in another study. In this study, CTLA-4 positivity was also higher in MAIT cells. Combined, it was proposed that MAIT cells were exhausted in AIH (24).

B cells, including memory and plasma cells, are involved in AIH humoral immunity (9). In our study, systemic total B cell frequency was similar at baseline and remained as such following IS. In the liver, B cell proportions were higher in AIH patients compared to controls. However, these samples were from cross-sectionally assessed patients. It was not possible to explore the role of IS on hepatic B cells. Nonetheless, several groups clearly demonstrated that, by IHC, intrahepatic B cell numbers and or proportions decline sharply following IS (10, 20, 22), which was even bigger compared to the CD4 and CD8 cell number reductions. This may cause to question of whether or not B cells are incumbent as a “driver” in the pathogenesis of AIH (25).

One of the novel findings of this study is the alteration in Bregs in the periphery and the liver in AIH/AISC. They are indispensable in the maintenance of tolerance and immune homeostasis, despite representing fewer than 10% of total B cells in the circulation in healthy individuals (26–29). Even more, numerical and/or functional impairments in CD24+CD38+ or CD24+CD27+ Bregs were also implicated in autoimmunity (rheumatoid arthritis, psoriasis, systemic sclerosis), viral infection (hepatitis B virus), and allergy (allergic rhinitis) (30). The involvement of Bregs in AIH is currently unknown (25). Nonetheless, in primary biliary cholangitis (PBC), it was demonstrated that the frequency of peripheral Bregs was significantly elevated in PBC patients compared to HCs, advocating against a numerical impairment (31). These Bregs expressed less T cell immunoglobulin mucin domain-1 (Tim-1), which has immunoregulatory properties. Previously, it was shown that a variant of it, Tim-3, was also downregulated in T cells in AIH (32). In our study, the proportion of peripheral Bregs at diagnosis demonstrated no difference compared to HCs. The CTLA-4+ proportion within Bregs was also unaltered. However, following the initiation of IS treatment, Bregs’ proportion decreased sharply in the prospective cohort and remained low in the longitudinal cohort. Hence, Breg homeostasis is deranged. Peripheral paucity was also reflected in the liver. The frequency of hepatic Bregs was lower compared to HC, HTs, and disease/treatment controls. Actually, this is not the first report of peripheral Bregs scarcity caused by IS treatment in AIH patients. Our group had the unique opportunity to monitor a child with recent onset of AIH who also developed COVID-19 disease (33). As we were screening the immune system for potential changes owing to this infection, we found that starting IS severely reduced Bregs in the blood, which did not recover during the observation period. The current investigation allowed us to confirm this phenomenon. Another study found that recipients of allografts with plasma cell hepatitis who received multiple IS also had significantly fewer Bregs and IL-10+ B cells in their blood than HCs (29). Furthermore, we also wondered whether a particular IS drug was to blame for the Bregs decline. In our Tac treated transplanted cohort, Bregs frequency was not much affected compared to our healthy controls and disease control (DILI). Our *in vitro* results did not particularly show a decline of Bregs frequencies after IS incubation of healthy PBMCs. Actually, the results from our *in vitro* data are in contradiction with our data where treatment with IS clearly caused a drop in Breg frequencies in all patients and at all time points. Bregs (transitional B cells) continue their maturation process in the secondary lymphoid organs such as the spleen and lymph nodes (26, 27, 29, 30). Furthermore, the homing of B cells between secondary lymphoid organs and (inflamed) tissues is regulated by chemokine receptors such as CXCR5, CCR7, CXCR3, CCR1, and CCR5 (34). Similarly, another study about Bregs in plasma cell hepatitis patients found that these cells express integrins (CD11a, CD11b, α1, α4, and β1) (29). It is possible that the expression of all these markers and thereby the homing capacity of Bregs are affected by IS, potentially explaining why Bregs in the blood almost disappeared. A recent study demonstrated that BCR signaling was affected following 4 hours of *in vitro* incubation with methylprednisolone (35). However, whether this also affected homing markers on B cells/Bregs requires further investigation. Additionally, we found that Tac and 6MP increased the frequency of IL-10+ B cells. Similar trends were also noted in IL-10+ Bregs. Yet, the aforementioned study also found that methylprednisolone stimulated the expression of IL-10 mRNA in B cells (35). It seems that IS drugs have an effect on B cell/Breg homeostasis and on their IL-10 production.

Lastly, we also found that not HLA-DR3, DR4, DR7, nor DR13, but HLA-DR11 is the most frequent allele in AIH patients. These results are not compatible with earlier studies in Caucasian AIH patients. However, this is not entirely surprising as the ancestry of the current Turkish population has roots mainly in central Asia. Yet, two Turkish studies investigated the HLA-DRB1 allele frequency in children or adult AIH patients (36, 37). In adults, they found that 58% of patients had DR4 with the DR3 frequency being similar to our population 29%. However, the study in children did find that HLA-DR11 was one of the most common alleles. However, mechanistically, it is still unknown why DR11 may be linked to AIH in Turkish patients. One intriguing avenue of investigation is computational modeling, which could shed light on HLA-DR11 characteristics such as autoantigen binding capacity and stability mediated by HLA-DM (38). For example, peptide binding is regulated by the charge and the three-dimensional structures of amino acids (AA) constituting the antigen-binding pockets in the peptide binding groove. One such pocket is formed by HLA-DRβ chain AAs between positions 67 and 72. These AA encode LLEQKR (DR3) and LLEQRR (DR4) motifs (5, 39, 40). Indeed, we found that HLA-DR11 expresses FLEDRR, which is 100% similar to DR3/DR4 based on the charge of the AAs. Additionally, the AA at position 71, lysine (K) or arginine (R), is paramount in conferring susceptibility to AIH as HLA-DR15 (protective against AIH) differs at AA position 71 encoding alanine (uncharged) (41). HLA-DR11 has also arginine at position 71, potentially explaining why HLA-DR11 is prevalent in our AIH cohort.

There are also limitations in our study. The sample size of the prospective AIH Group-1 is small owing to the fact that AIH is a very rare disease. The serial assessment of AIH livers before and during IS was not always possible because the initial diagnosis and commencement of IS may have already started by the time patients were referred to our tertiary center. Furthermore, children donate a limited amount of blood. By virtue, functional assays such as the baseline and/or *in vitro* IL-10 production in Bregs and total B cells following IS could not be performed in these patients alongside deep peripheral immune phenotyping. Instead, we used age-matched HCs. Lastly, there are several Breg populations such as CD20+CD24^hi^CD27+ memory B cells and CD20^low^CD38+CD27^low^ plasmablasts among others (26). Their individual susceptibility to IS is unknown, which is a matter for further investigation. In adults, they found that 58% of patientshad DR4 with the DR3 frequency being similar to our population 29%. However, the study in children did find that HLA-DR11 was one of the most common alleles.

In conclusion, major findings in the peripheral blood of naïve AIH patients were: fewer activated Tregs and MAIT cells with preserved Breg frequencies. Following IS, most cell frequencies fluctuated, with total and naïve Tregs decreasing over time. Even more, the proportion of Bregs dropped sharply. The paucity of Bregs was also observed in the livers. However, *in vitro* IS did not affect the proportion of Bregs but increased IL-10+ total B cells, instead. Importantly, the current findings of Bregs scarcity need to be investigated to comprehend what the potential clinical implications (good response to IS, survival, and liver transplantation) are. Further research into the role of DR11 in AIH is also warranted in larger AIH cohorts that include local health subjects.

## Data availability statement

The original contributions presented in the study are included in the article/**Supplementary Material**. Further inquiries can be directed to the corresponding author.

## Ethics statement

The studies involving human participants were reviewed and approved by Koç University. Written informed consent to participate in this study was provided by the participants’ legal guardian/next of kin.

## Author contributions

ÇA and MY participated in the study conception and design. ÇA, MY, FN, EP, and DW were involved in the acquisition of samples, analysis, and interpretation of data. ÇA, MY, FN, and MA drafted the manuscript, and also provided critical revisions. SA overviewed HLA DRB1 allele typing. All authors contributed to the article and approved the submitted version.

## Supplementary material

Supplementary Figure 1 Schematic overview of the different groups, time points, and tissues assessed in this study **(A)** Shows the assessment time points for the prospective cohort of naïve AIH patients. **(B)** Shows the different groups used as disease control, treatment control, or healthy control.

Supplementary Figure 2 Gating strategy for all immune cells and their subsets. This figure sets out in detail all parent gates and used CD markers defining the mentioned cells and their subsets, starting from FSC and SSC lymphocyte gating. Specifically, we show gating for total B cells, total T cells, CD4 and CD8 T cells, memory B cells and Bregs. Within the Bregs, we also gate for CTLA-4. Furthermore, naïve CD4 and CD8 T cells are shown. Both naïve and activated Tregs are gated as well with CTLA-4 gating for activated Tregs. Activated Tregs are also gated for their CD25 expression. Lastly, the gating strategy for NK and NKT cells as well as MAIT cells, are provided.

Supplementary Figure 3 Analysis of ratios of effector T cells to total Tregs. **(A)** Is a bar chart of the ratio of effector T cells to total Tregs in healthy control and AIH patients before or following treatment.

Supplementary Figure 4 Comparisons between autoimmune sclerosing cholangitis and patients with autoimmune hepatitis **(A)** Is a bar chart of the frequency of peripheral blood immune cells such as total T cells, total B cells, CD4 T cells, CD8 T cells, naïve (CD45RA+) CD4 and CD8 T cells, and memory B cells. **(B)** Demonstrates the proportion of total, naïve, and activated (with or without CTLA-4) Tregs. **(C)** Provides frequencies of NK/NKT cells and MAIT cells. **(D)** Is a bar chart of Bregs.

Supplementary Figure 5 Analysis of ratios of effector T cells to total Tregs in healthy livers, healthy liver tissue, autoimmune sclerosing cholangitis, and patients with autoimmune hepatitis. **(A)** Is a bar chart of the ratio of effector T cells to total Tregs in the livers.

Supplementary Figure 6 Flow cytometry dot plots of total B cells and Bregs producing IL-10. **(A, B)** demonstrates gating for total B cells and Bregs. **(C, D)** shows gating for B cells and Bregs producing IL-10 in healthy controls under various *in vitro* (immunosuppressive) conditions. **(E, F)** are FMOs for total B cells and Bregs, respectively.

Supplementary Figure 7 Analysis of HLA-DRB1 allele distribution. **(A)** Demonstrates the HLA-DRB1 allele frequency in AIH-1. **(B)** Demonstrates the HLA-DRB1 allele frequency in AIH-2.
