# Supplementary figures and images for: Standard immunosuppressive treatment reduces regulatory B cells in children with autoimmune liver disease
Source: Front Immunol. 2023 Jan 5;13:1053216. doi: 10.3389/fimmu.2022.1053216 (PMC9849683; doi:10.3389/fimmu.2022.1053216)

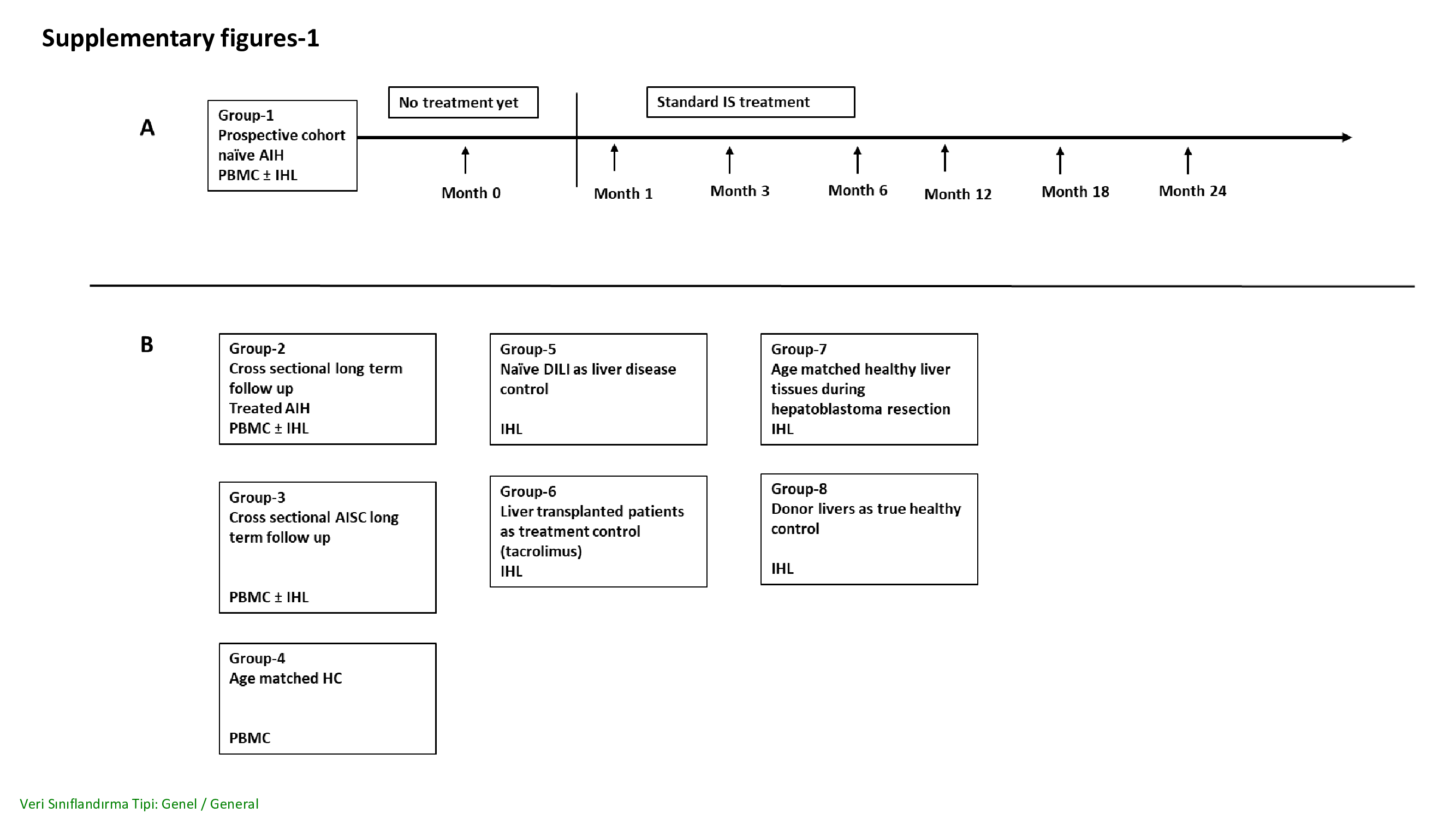

Supplement: Supplementary Figure 1 — Schematic overview of the different groups, time points, and tissues assessed in this study (A) Shows the assessment time points for the prospective cohort of naïve AIH patients. (B) Shows the different groups used as disease control, treatment control, or healthy control. [file Image_1.png]

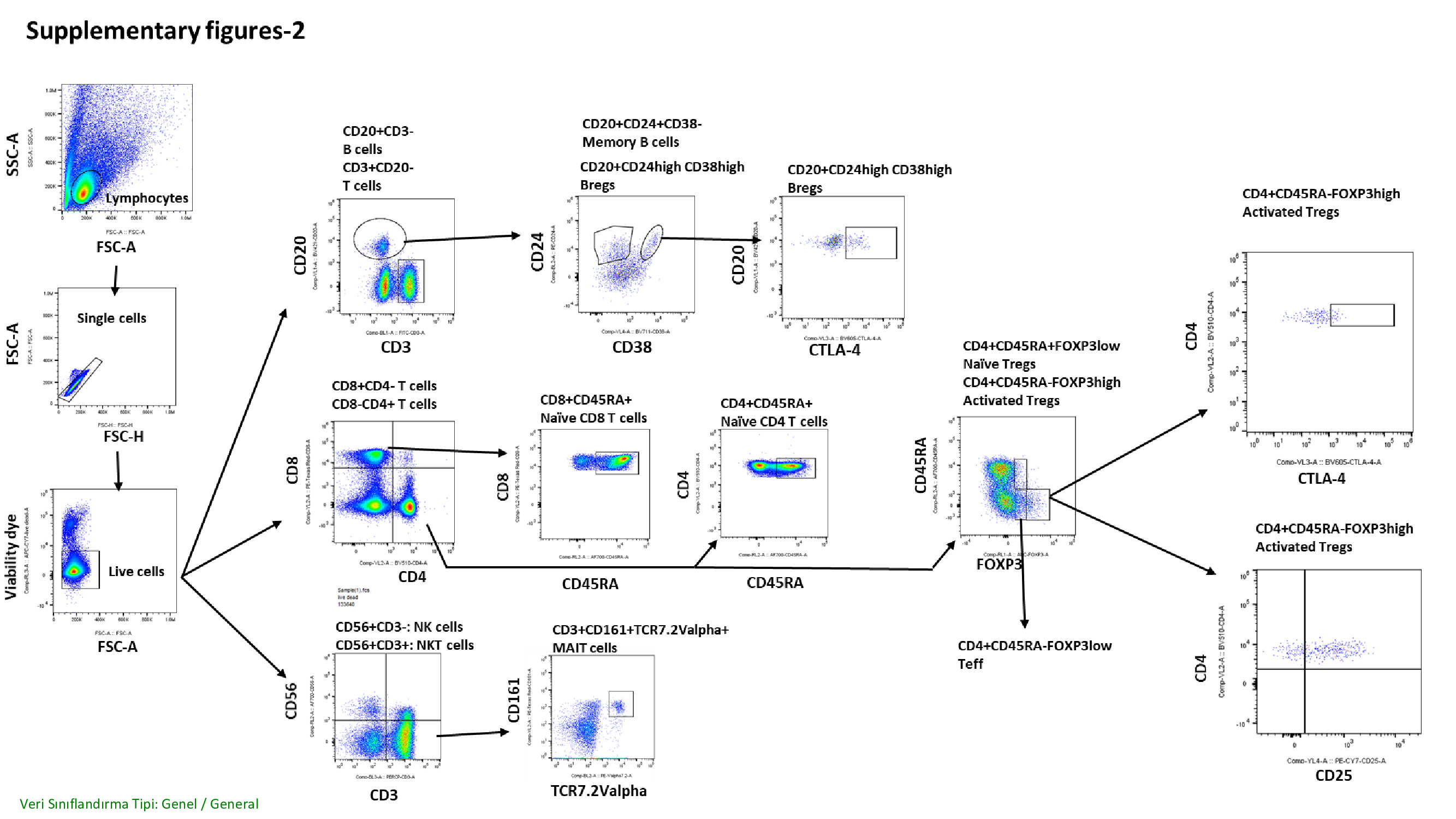

Supplement: Supplementary Figure 2 — Gating strategy for all immune cells and their subsets. This figure sets out in detail all parent gates and used CD markers defining the mentioned cells and their subsets, starting from FSC and SSC lymphocyte gating. Specifically, we show gating for total B cells, total T cells, CD4 and CD8 T cells, memory B cells and Bregs. Within the Bregs, we also gate for CTLA-4. Furthermore, naïve CD4 and CD8 T cells are shown. Both naïve and activated Tregs are gated as well with CTLA-4 gating for activated Tregs. Activated Tregs are also gated for their CD25 expression. Lastly, the gating strategy for NK and NKT cells as well as MAIT cells, are provided. [file Image_2.png]

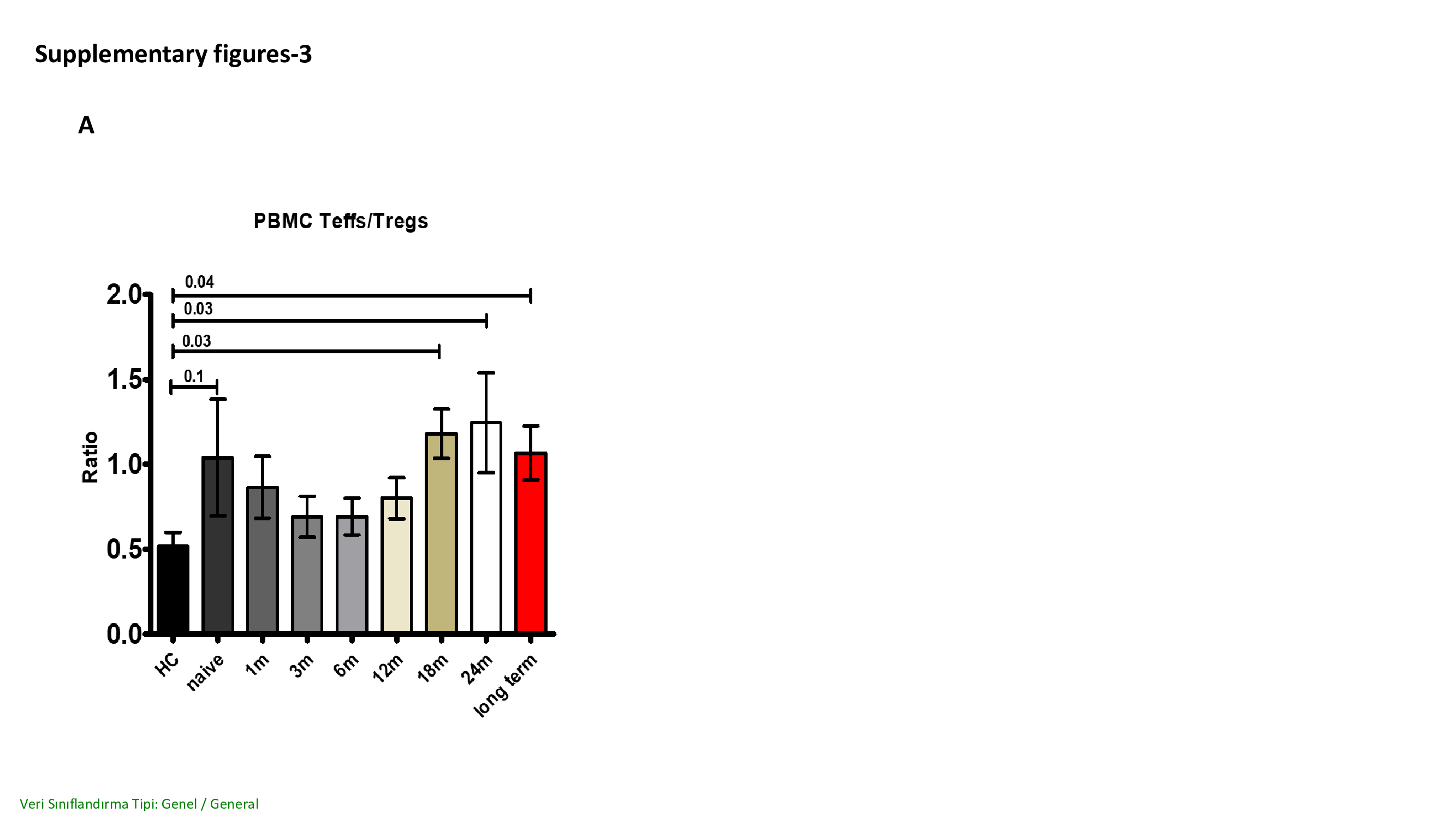

Supplement: Supplementary Figure 3 — Analysis of ratios of effector T cells to total Tregs. (A) Is a bar chart of the ratio of effector T cells to total Tregs in healthy control and AIH patients before or following treatment. [file Image_3.png]

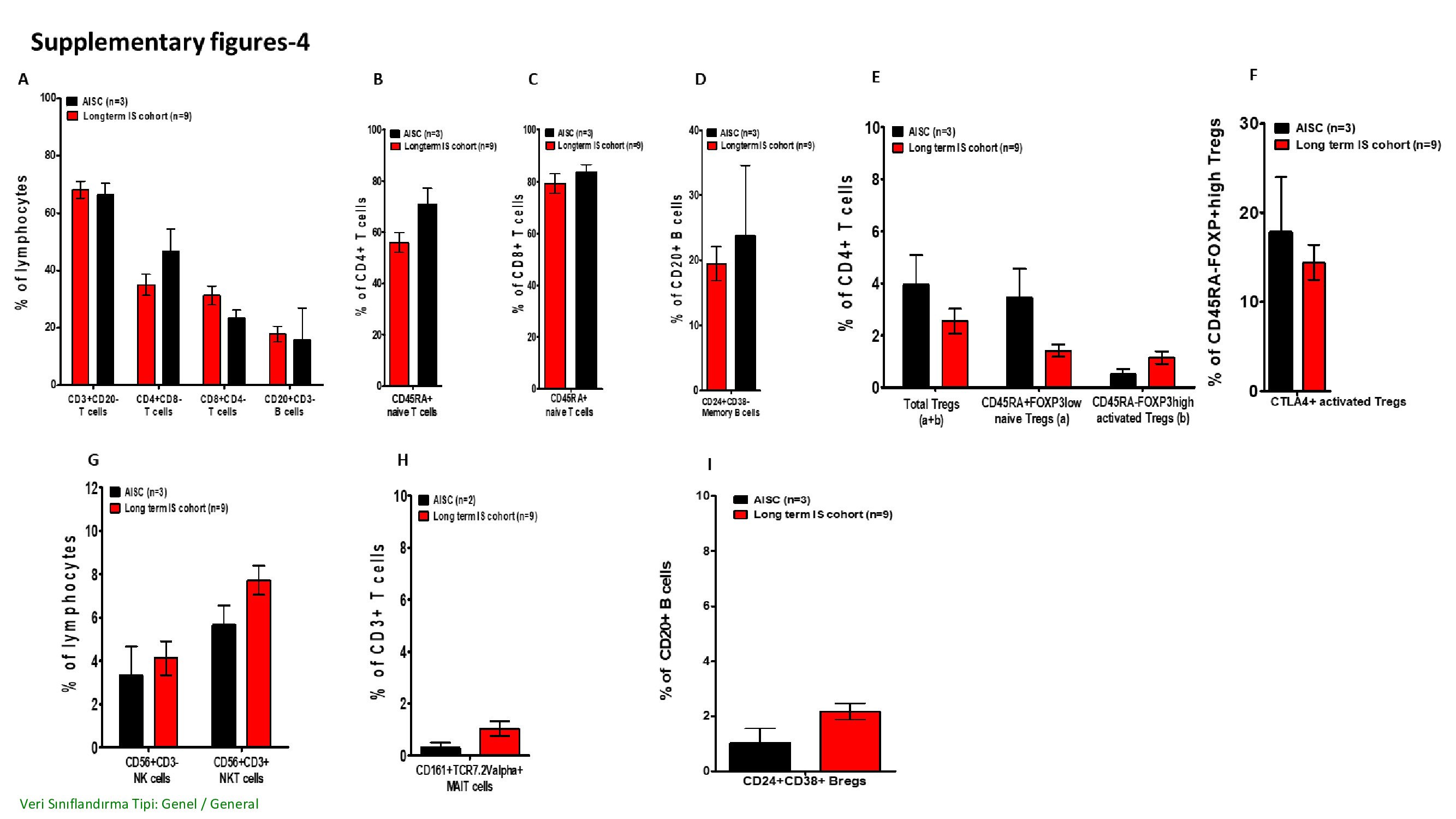

Supplement: Supplementary Figure 4 — Comparisons between autoimmune sclerosing cholangitis and patients with autoimmune hepatitis (A) Is a bar chart of the frequency of peripheral blood immune cells such as total T cells, total B cells, CD4 T cells, CD8 T cells, naïve (CD45RA+) CD4 and CD8 T cells, and memory B cells. (B) Demonstrates the proportion of total, naïve, and activated (with or without CTLA-4) Tregs. (C) Provides frequencies of NK/NKT cells and MAIT cells. (D) Is a bar chart of Bregs. [file Image_4.png]

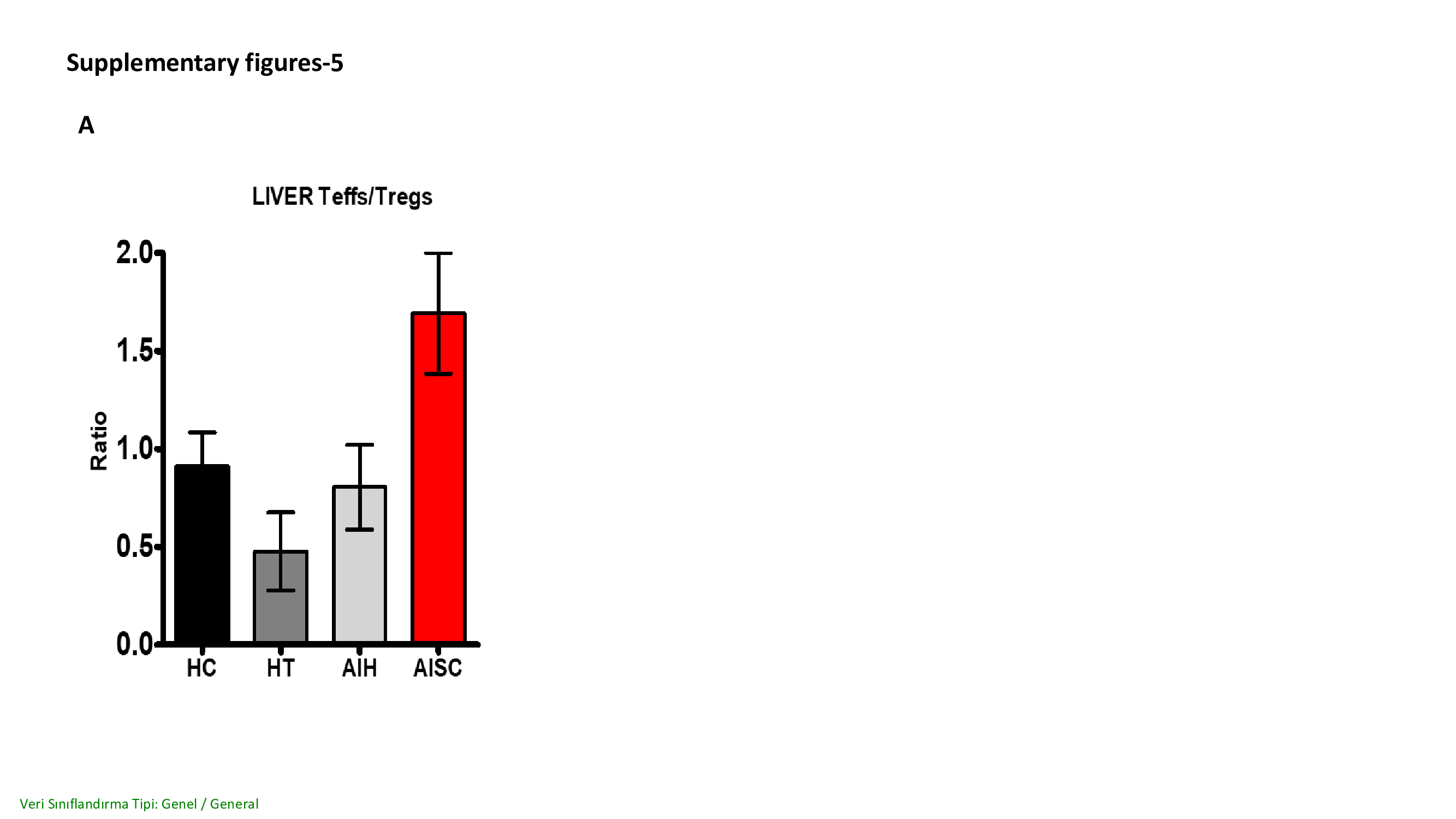

Supplement: Supplementary Figure 5 — Analysis of ratios of effector T cells to total Tregs in healthy livers, healthy liver tissue, autoimmune sclerosing cholangitis, and patients with autoimmune hepatitis. (A) Is a bar chart of the ratio of effector T cells to total Tregs in the livers. [file Image_5.png]

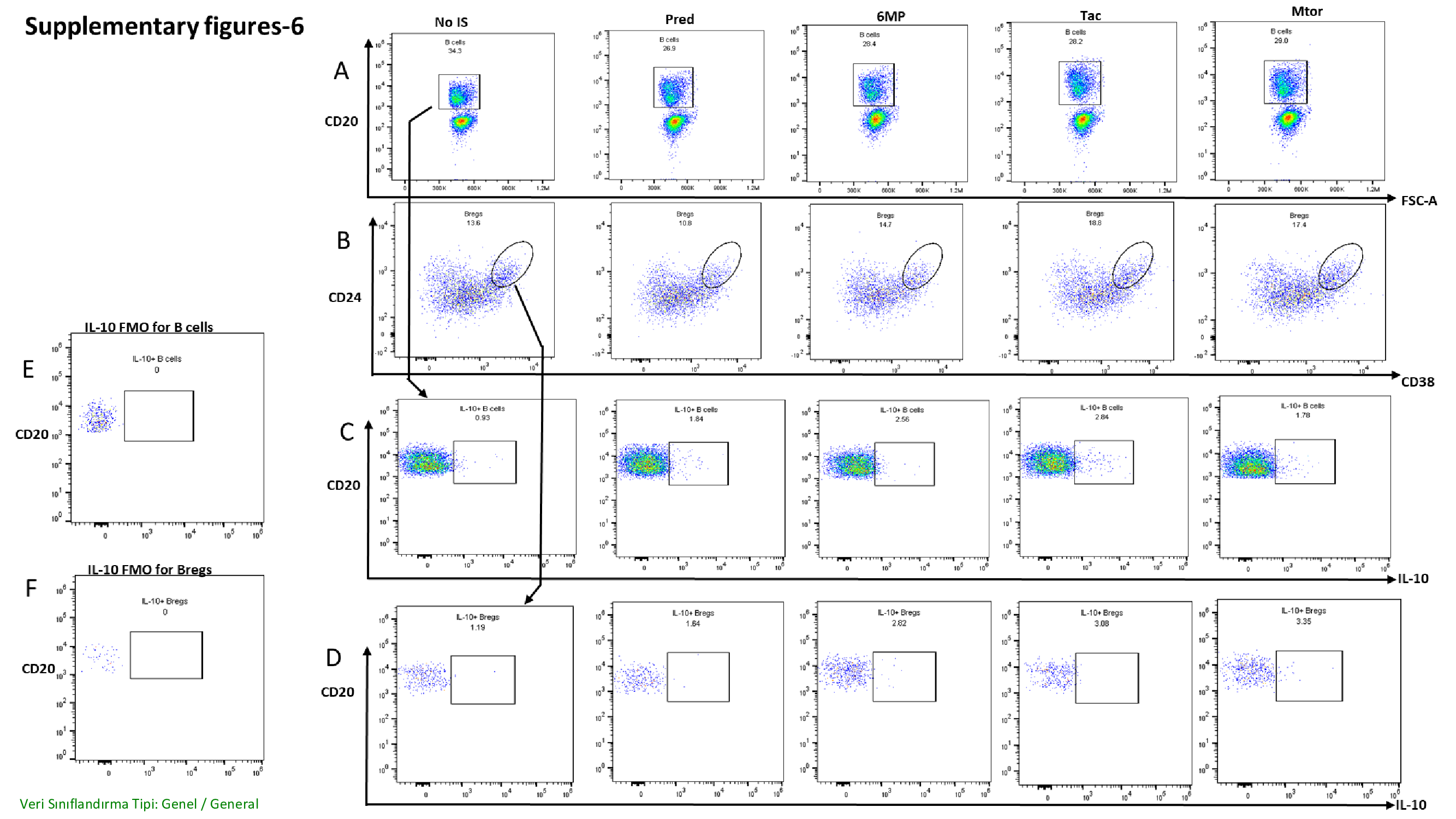

Supplement: Supplementary Figure 6 — Flow cytometry dot plots of total B cells and Bregs producing IL-10. (A, B) demonstrates gating for total B cells and Bregs. (C, D) shows gating for B cells and Bregs producing IL-10 in healthy controls under various in vitro (immunosuppressive) conditions. (E, F) are FMOs for total B cells and Bregs, respectively. [file Image_6.png]

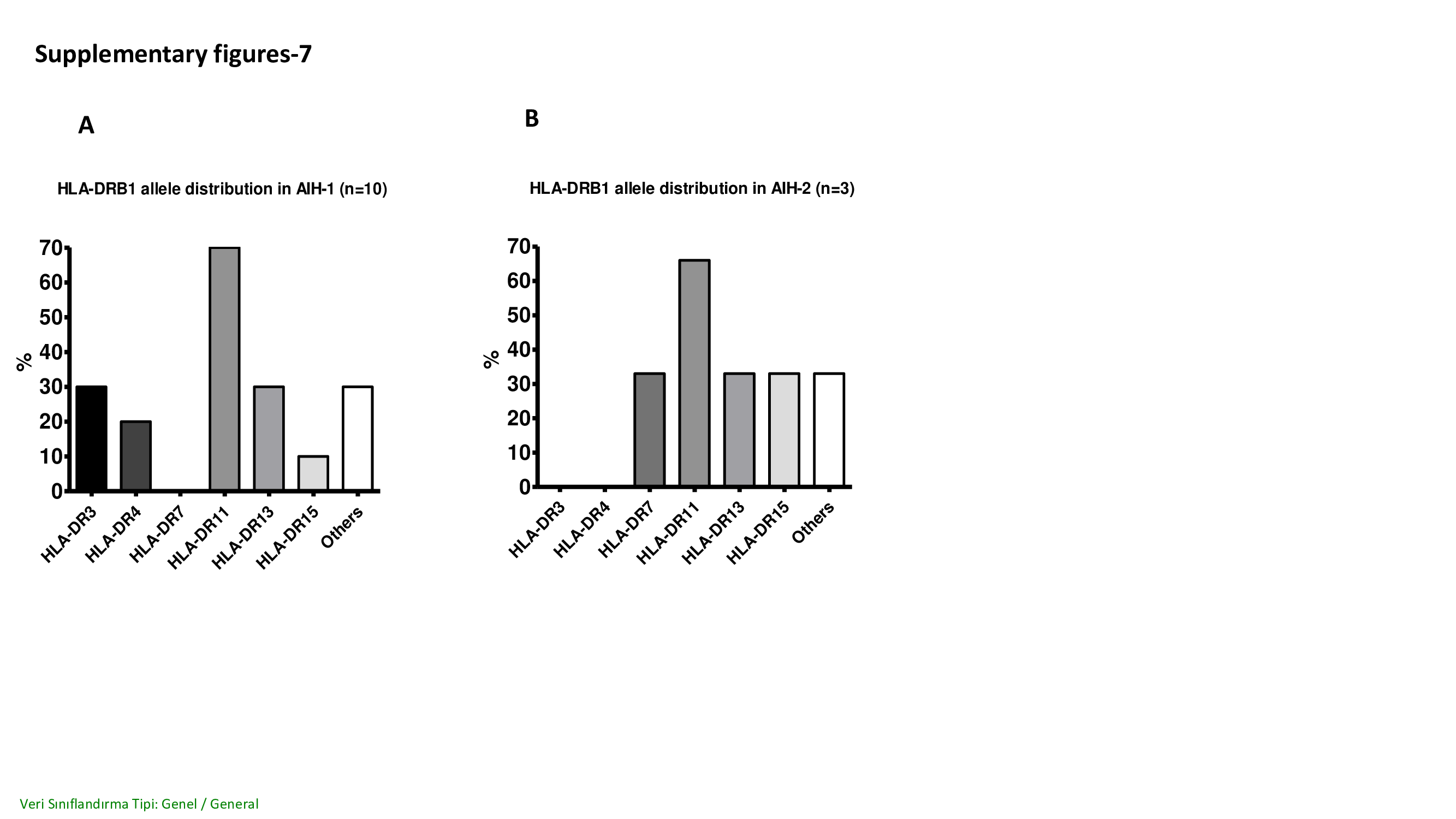

Supplement: Supplementary Figure 7 — Analysis of HLA-DRB1 allele distribution. (A) Demonstrates the HLA-DRB1 allele frequency in AIH-1. (B) Demonstrates the HLA-DRB1 allele frequency in AIH-2. [file Image_7.png]
